# Towards two-dimensional color tunability of all-solid-state electrochromic devices using carbon dots

**DOI:** 10.3389/fchem.2022.1001531

**Published:** 2022-08-30

**Authors:** Chen Li, Mingshuo Zhen, Boshan Sun, Yingping Hong, Jijun Xiong, Wenzhi Xue, Xiaohua Li, Zhongkun Guo, Lei Liu

**Affiliations:** ^1^ Science and Technology on Electronic Test and Measurement Laboratory, North University of China, Taiyuan, China; ^2^ State Key Laboratory of Dynamic Measurement Technology, North University of China, Taiyuan, China; ^3^ Key Laboratory of Instrumentation Science & Dynamic Measurement, Ministry of Education, North University of China, Taiyuan, China; ^4^ School of Energy and Power Engineering, North University of China, Taiyuan, China

**Keywords:** electrochromism, carbon dots, multicolor patterns, high temperature environments, synergistic effect

## Abstract

Electrochromic devices (ECDs) that display multicolor patterns have gradually attracted widespread attention. Considering the complexity in the integration of various electrochromic materials and multi-electrode configurations, the design of multicolor patterned ECDs based on simple approaches is still a big challenge. Herein, it is demonstrated vivid ECDs with broadened color hues via introducing carbon dots (CDs) into the ion electrolyte layer. Benefiting from the synergistic effect of electrodes and electrolytes, the resultant ECDs presented a rich color change. Significantly, the fabricated ECDs can still maintain a stable and reversible color change even in high temperature environments where operating temperatures are constantly changing from RT to 70°C. These findings represent a novel strategy for fabricating multicolor electrochromic displays and are expected to advance the development of intelligent and portable electronics.

## Introduction

Electrochromic devices (ECDs) have attracted increased attention in smart windows, displays and color-tuneable optical elements because of their zero energy consumption when at various working states (Ma et al., 2021; Kim et al., 2021; Cui et al., 2019; Seong et al., 2018; Park et al., 2017; Wu et al., 2021). Especially, multicolor electrochromic displays are one of the most versatile applications because they can retain their colored states without the need to supply electrical power. To date, ECDs based on polymers, organic molecules and metal-organic frameworks have demonstrated multicolor characteristics, while these materials possess inferior chemical and thermal stabilities compared to inorganic electrochromic materials. These shortcomings seriously hinder their real-world applications and potential commercialization. In this regard, inorganic multicolor ECDs are regarded as a paradigm shift in the field of electrochromic displays. However, inorganic electrochromic materials exhibit limited colors. For example, WO_3_ has good long-term stability and is often used as a cathode material for ECDs, but it can only display a blue state (Yan et al., 2014; Bo et al., 2019). NiO, the anode material for ECDs, also has only a black-brown state (Phan et al., 2021). Zhang et al. (2021) achieved multicolor response by constructing a three-electrode system (one reference electrode, two working electrodes) to colorize the cathode and anode of ECDs respectively. However, ECDs with three-electrode structure are not only difficult to fabricate, but the electrochemical performance of ECDs will inevitably decrease. Therefore, rich and subtle color adjustment has rarely been achieved by inorganic electrochromic materials incorporating these modifications, and this limitation has already become a bottleneck for the further development of electrochromic technology. Given this bottleneck, it is crucial to develop novel structures for inorganic ECDs to broaden their color palettes.

Generally, traditional ECDs are composed of five layers, including transparent conductive layer, electrochromic layer, ionic conductor layer, ion storage layer and transparent conductive layer (Thakur et al., 2012). Most of the electrochromic layer and ion storage layer are transition metal oxides, and the color of transition metal oxides is relatively limited (Bueno et al., 2008; Ma et al., 2021; Tang et al., 2021). Consequently, it is a feasible strategy to design multi-color ECDs from the perspective of electrolyte layer (Périé et al., 2022; Chang et al., 2018; Kanazawa and Uemura, 2019; Chan et al., 2021). As a fluorescent labeling material, carbon dots (CDs) can display various colors by controlling their concentration in organic solvents (Wang et al., 2020; Ma et al., 2020; Wang et al., 2021; Wei et al., 2022). In this work, a novel concept for transparent inorganic multicolor ECD was successfully designed and fabricated by introducing CDs into the ion electrolyte layers. Different from conventional ECDs, this novel ECD incorporates different amounts of CDs in the solid-state electrolytes (PC-LiClO_4_). Through adjusting the content of CDs, ECDs can not only exhibit rich color changes but also improve their electrochemical stability. Significantly, the fabricated ECD can still maintain a stable and reversible color change even in harsh environments where operating temperatures are constantly changing from RT to 70°C (Lei et al., 2019; Liu et al., 2021; Wang et al., 2022). We envision that our as-obtained ECDs with large charge capacity, multicolor display and high cycle stability can be promising for future color switching/energy storage applications and may also provide new insights into the design of multifunctional electronics.

## Experimental

The substrate used in the experiment is conductive ITO glass (30 mm × 35 mm), and the dirt on the surface shall be cleaned with acetone, ethanol and deionized water respectively before deposition. After cleaning, dry it with a blower for standby.

### Deposition of manganese dioxide thin films for anode materials

An electronic balance weighed 2.84 g of sodium sulfate and 4.90 g of manganese acetate, dissolved them in 200 ml of deionized water, and continued magnetic stirring until they were completely dissolved to obtain a manganese dioxide deposition solution.

In this experiment, an electrochemical workstation (CORRTEST: CS2350H) was used for electrochemical deposition, three electrode system is adopted for deposition. Ag/AgCl was used as the reference electrode, platinum electrode was used as the counter electrode, and the above-cut ITO glass was used as the working electrode, and the electrolyte was the above-configured manganese dioxide deposition solution was polarized with a constant current of 2.5 mA for 180 s to obtain a manganese dioxide film (Wang et al., 2020). The as-prepared MnO_2_ electrodes were placed into a tube furnace and heated at 200°C in high-purity Ar for 1 h. In addition, the detailed heat treatment curves about them are presented in Supplementary Figure S1. XPS survey spectrum was investigated to demonstrate the existence of MnO_2_ and show the chemical composition, which all peaks of Mn, C, O elements are clearly observed after calibrating the peak of C1s (Supplementary Figure S2A). After charging, the amount of Mn^4+^, Mn^3+^ and Mn^2+^ are about 59.6%, 32.1%, and 8.3%, respectively (Supplementary Figure S2B **, bottom**). After discharging, the amount of Mn^4+^, Mn^3+^ and Mn^2+^ are about 33.8%, 40.5%, and 25.7%, respectively (Supplementary Figure S2B **, top**).

Subsequently, the thickness of the MnO_2_ was measured, and the thickness of the MnO_2_ before and after the heat treatment was about 350 nm (Supplementary Figure S3).

### Deposition of prussian blue film of cathode material

The electronic balance weighs 0.32 g of anhydrous ferric chloride, 0.66 g of potassium ferricyanide and 1.5 g of potassium chloride, dissolves them in 200 ml of deionized water, continues magnetic stirring until they are completely dissolved, and then adds concentrated sulfuric acid to adjust the pH to one to obtain the deposition solution for electroplating Prussian blue.

Prussian blue also adopts a three electrode system, with Ag/AgCl as the reference electrode, platinum electrode as the counter electrode, and the above cut ITO glass as the working electrode, in which the electrolyte is the above configured Prussian blue solution, and the Prussian blue film is deposited by constant current polarization at a potential of −0.16 mA for 360 s (Feng et al., 2010).

### Preparation of carbon dots

Weigh 200 mg of coal tar powder with an electronic balance, add it to a mixed solution of 30 ml of formic acid and 3 ml of H_2_O_2_, stir at room temperature for 20 h, and then centrifuge the etched and oxidized solution at 10,000 r/min for 15 min. Take out the supernatant containing Coal-based CDs, discard the unreacted coal tar pitch, and finally spin the supernatant containing Coal-based CDs on a vacuum rotary evaporator to obtain rough Coal-based CDs powder. The rough coal-based CDs were dissolved in a solution of 50% water and 50% ethanol, and then packaged with a dialysis bag and put into a large beaker containing deionized water. The large beaker was put into a water bath and boiled at 400 r/min for 10 min at 110°C. And then the coal-based CDs powder was obtained after drying in the blast drying oven. (Singamaneni et al., 2015). The TEM image of the prepared carbon dots is shown in Supplementary Figure S4.

### Preparation of electrochromic devices with carbon dots

**FIGURE 1:**
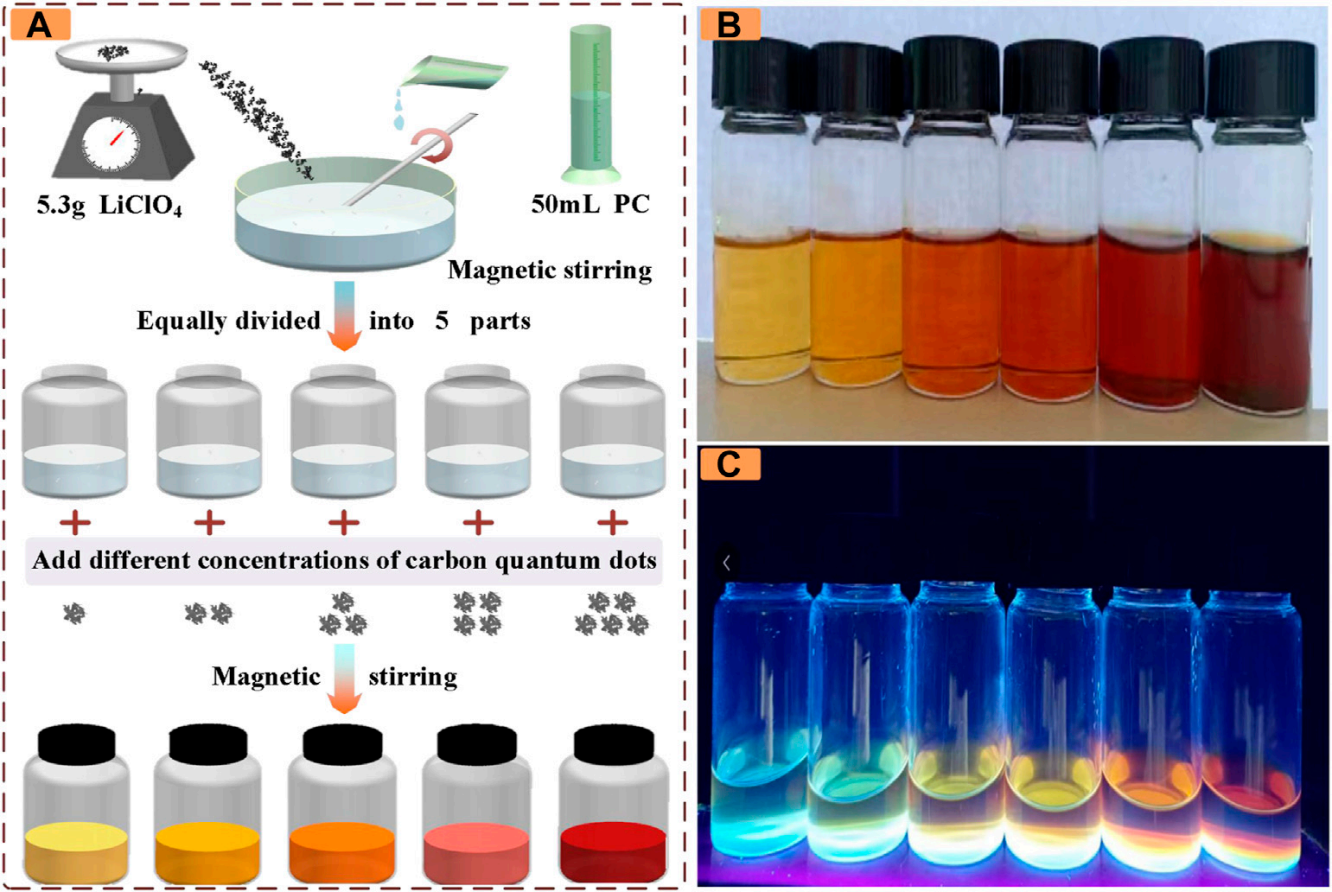
**(A)** Preparation process of electrolyte solution **(B)** The colors under different CDs masses are 1, 2, 4, 8, 12, and 20 mg from left to right under visible light **(C)** The color of different CDs masses under ultraviolet light is 1, 2, 4, 8, 12, and 20 mg from left to right.

In this study, PC/LiClO_4_ electrolyte containing CDs is used as the ionic conductor layer of ECDs. Figure 1A shows the preparation process of electrolyte. As shown in Figure 1A, in step 1, prepare 50 ml of 1 mol/L PC-LiClO_4_ solution. Step 2: evenly divide the above prepared solution into five parts. In step 3, CDs of different masses are added to the prepared solution and stirred for 10 min to obtain electrolyte solutions of different colors. To prepare the ECDs with CDs, the electrolyte with CDs was sandwiched between a manganese dioxide electrode and a Prussian blue electrode, followed by the irradiation of 365 nm ultraviolet light with an intensity of 23 mW cm−^2^ for 15 min to induce the electrolyte curing. After curing, ECDs with CDs can be obtained.

### Electrochemical and electrochromic performances measurements

Cyclic voltammetry (CV), chronoamperometry (CA) and galvanostatic charge/discharge (GCD) properties of the ECDs were studied using an electrochemical workstation. All electrochromic measurements in this work were carried out by a UV–vis spectrophotometer (PUXI: TU-1901) coupling with above electrochemical workstation, from which *insitu* absorption and transmittance spectra of the ECDs could be obtained. The ECD was fixed to a ceramic heating chip that contained holes for the high-temperature measurements. Further details are presented in Supplementary Figure S5.

## Results and discussion

### Performance analysis of electrolyte solution doped with carbon dots

As shown in Figure 1B, the concentration of the prepared electrolyte solution is from left to right, and the color gradually changes from light yellow to orange and finally to red. In addition, due to the fluorescence effect of CDs in the UV band, its color was observed when the wavelength was 365 nm, as shown in Figure 1C. In the UV band, its color gradually changed from blue, yellow, brown to black. It provides a theoretical basis for the subsequent study of electrochromic properties of devices in the UV band.

**FIGURE 2:**
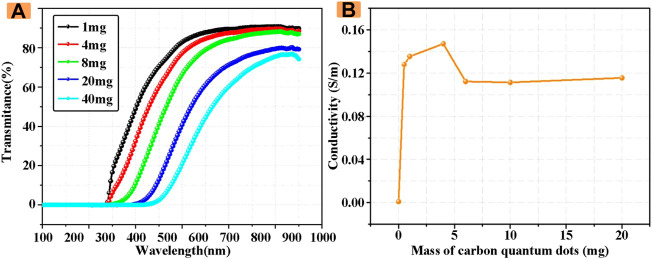
**(A)** Transmittance of electrolyte solutions with different CDs concentrations **(B)** Conductivity of electrolyte solutions with different CDs concentrations.

In order to better characterize the optical properties of electrolyte solution, its transmittance was measured by UV-Vis photometer. As shown in Figure 2A, with the increase of the concentration of CDs in the electrolyte solution, the transmittance of the electrolyte solution at the wavelength of 200–900 nm gradually decreased. The transmittance of the electrolyte solution doped with 1 mg of CDs at the wavelength of 200–900 nm was the highest at 90.4%. When the masses of CDs reach 40 mg, the highest transmittance is 76.7%, which is only 84% of the transmittance of 1 mg CDs introduced.

Subsequently, the conductivity of electrolyte solutions with different concentrations of CDs was studied in detail. The measurement results are shown in Figure 2B. When the concentration of CDs is 0, the conductivity is the minimum, and with the increase of the concentration of CDs, the conductivity increases and then decreases, and finally tends to be stable, and the conductivity that tends to be stable is still higher than that without CDs. Therefore, the doping of CDs can effectively increase the conductivity of electrolyte solution, and the increase of conductivity also indirectly improves the performance of ECDs, which also provides a theoretical basis for the subsequent study of the improvement of the performance of ECDs by CDs.

### Characterization of electrochromic devices

**FIGURE 3:**
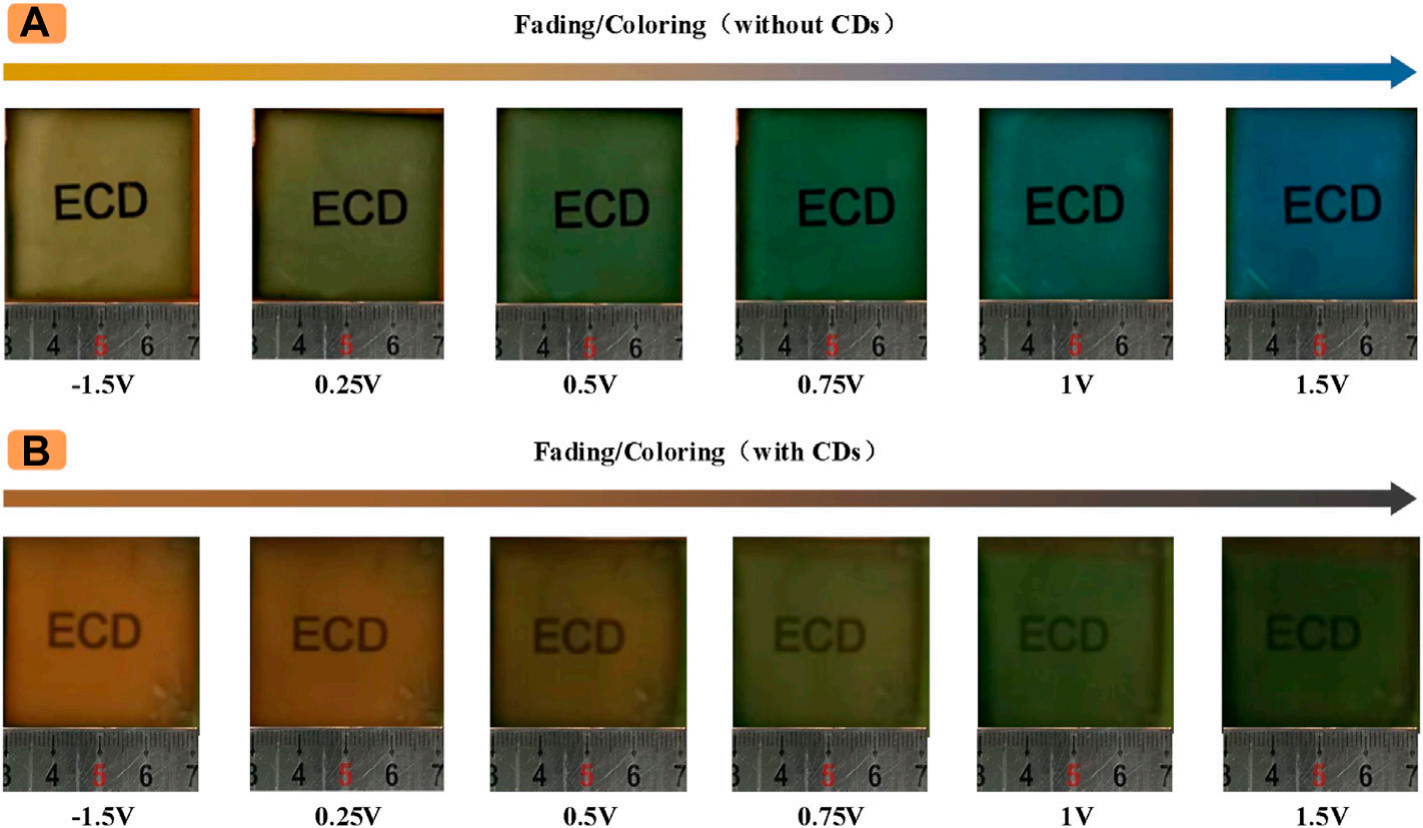
Color of ECDs at different potentials **(A)** Without CDs **(B)** With CDs.

For the convenience of comparison, we investigate the color of the ECD when the electrolyte solution was incorporated with and without CDs at different potentials. Which produced some other colors in the electrolyte solution compared to conventional ECDs. Traditional ECDs color changes with the potential change as shown in Figure 3A, in the initial state, the traditional ECDs fade state for light yellow, with the gradual increase of the positive voltage, blue deepening, devices become green at 0.5 V, continue to increase the positive voltage, finally at 1.5 V, the whole device to blue. The new ECD based on CDs shows other colors, as shown in Figure 3B, the device is orange-red in the initial state (0 V), and the ECD changes with the increasing forward voltage. The device slowly enters the coloring state, and the color gradually realizes the transformation of orange-red--orange--light yellow--yellow-green--dark green.

**FIGURE 4:**
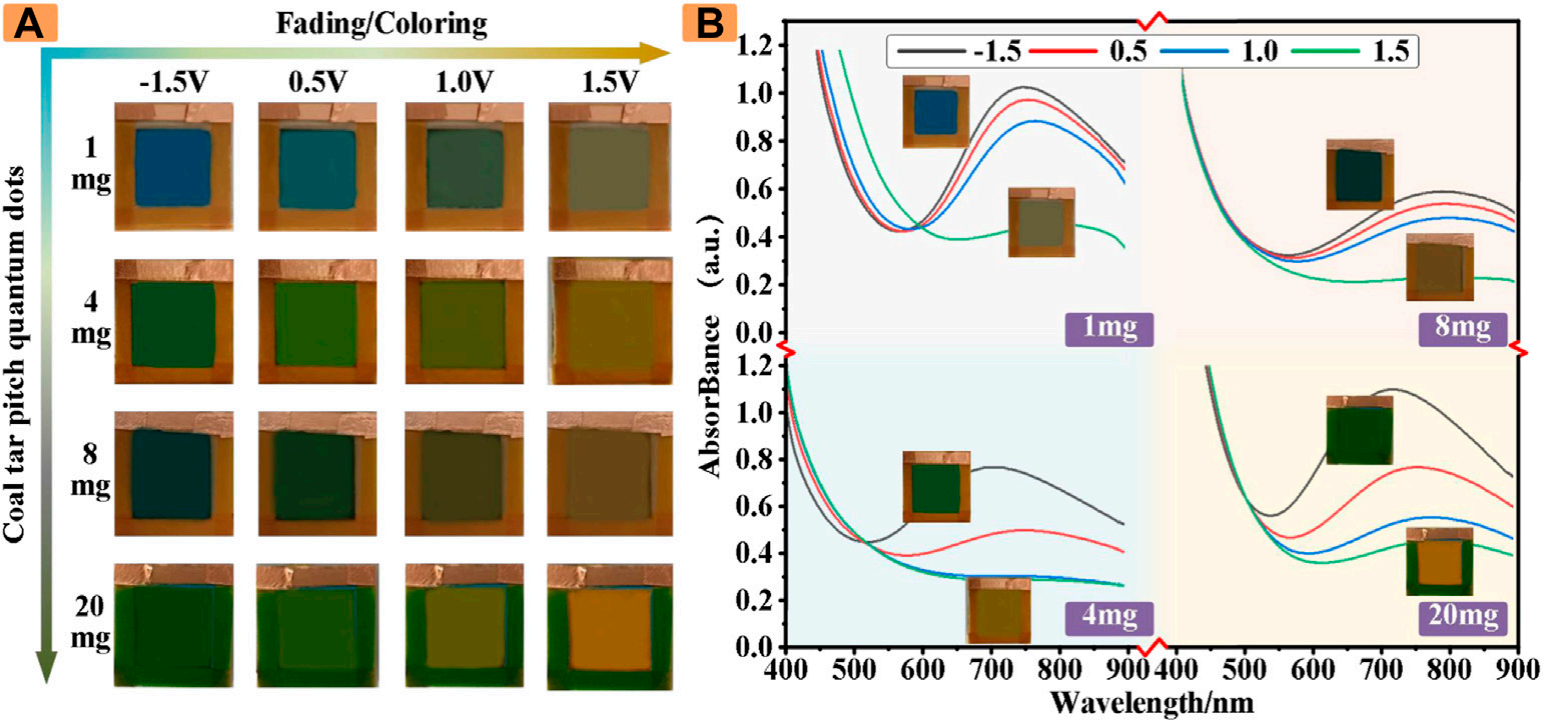
**(A)** The colors of ECDs with different masses of CDs at different voltages **(B)** The absorbance of ECDs with different masses of CDs.

Interestingly, the ECDs revealed rich and real-time color changes during the charge−discharge process. Figure 4A shows the optical images of the four different CDs concentrations of electrostatic discoloration devices, the initial color of the four ECDs are different. In the process of changing the voltage across the cathode and anode, the color of the ECD introduced with CDs changes correspondingly to produce a large number of polychromatic states, resulting in an almost blue, green to yellow color palette.

In addition to be perceived by the naked eye, these rich colors can also be analyzed quantitatively by the absorption spectrum of the UV-visible photometry. As shown in Figure 4B, these results demonstrated that our ECDs with CDs have a good multicolor effect and can be used as indicators of some colors.

**FIGURE 5:**
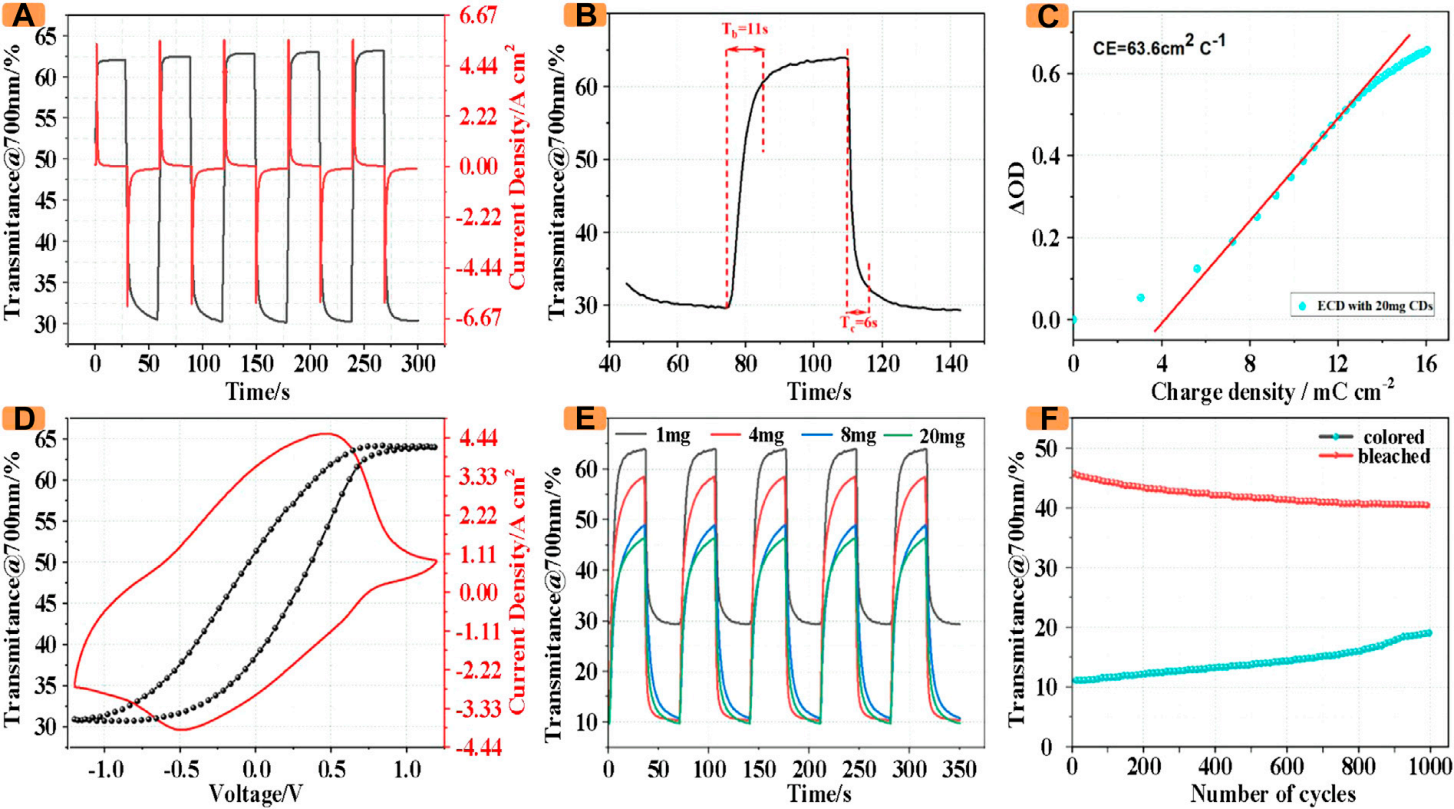
**(A)** CA and *in-situ* transmittance profile. **(B)** Response time for the switching between coloration and bleaching states. **(C)** Optical density vs. charge density curve. **(D)** CV and *in-situ* transmittance profile. **(E)** Transmittance of ECDs with different masses of CDs **(F)** Optical stability of 1,000 cycles at 700 nm.

Furthermore, the ECD incorporating CDs maintained good optical and electrochemical properties, as shown in Figure 5A, the ECD incorporating 1 mg CDs achieved an optical modulation amplitude of 32.8% at 700 nm. Simultaneously, the transmittance varies from 30.2% to 63%, and the ECD incorporating CDs has a coloration/bleaching switching time of 11 s and 6 s (Figure 5B).

To investigate the switching time and kinetics behavior, the chronoamperometry curve and the square-wave potential between −1.2 and 1.2 V as well as the *in-situ* transmittance spectra were recorded though combining a UV–vis spectrophotometer and an electrochemical workstation. Notably, both optical and current signals are indicated that the ECD with CDs possesses a fast response kinetics behavior. Moreover, as shown in Figure 5C, the ECD with CD_S_ obtains a coloration efficiency (CE) value of 63.6 cm2°C−^1^.

In order to investigate the relationship between the color change and the potential change of ECDs, the *in-situ* optical transmittance was recorded during the electrochemical test. As shown in Figure 5D, the ECD gradually transitioned from green to orange, accompanied by a sharp change in transmittance from 63% to 32.5%. On the contrary, when a negative voltage is applied, the transmittance reaches a minimum of 32.5% due to the superposition of the colors of the two electrodes and the electrolyte solution.

In order to more conveniently compare the optical properties of ECDs with CDs of different masses, we investigated the *in-situ* transmittance of the devices containing CDs of different masses at a wavelength of 700 nm. As shown in Figure 5E, with the increase of CDs, the transmittance of ECDs in coloring decreases. The transmittance of ECDs with 20 mg CDs in coloring is only 10%, which is only 33% of that with 1 mg CDs. At the same time, the transmittance of ECDs with 1 mg CDs in fading can reach 64%, while the transmittance of ECDs with 20 mg CDs in fading is only 69.2% of that with 1 mg CDs.

Furthermore, the cycling performance of the ECD with CDs was investigated by alternating application of a forward voltage (1.2 V) and reverse voltage (−1.2 V) at an interval of 30 s. As illustrated in Figure 5F, in the first cycle, the contrast ratio between the colored state and the faded state of the device was about After 1,000 cycles, the value is still 57.1% of the initial value, indicating that the ECD has good stability.

**FIGURE 6:**
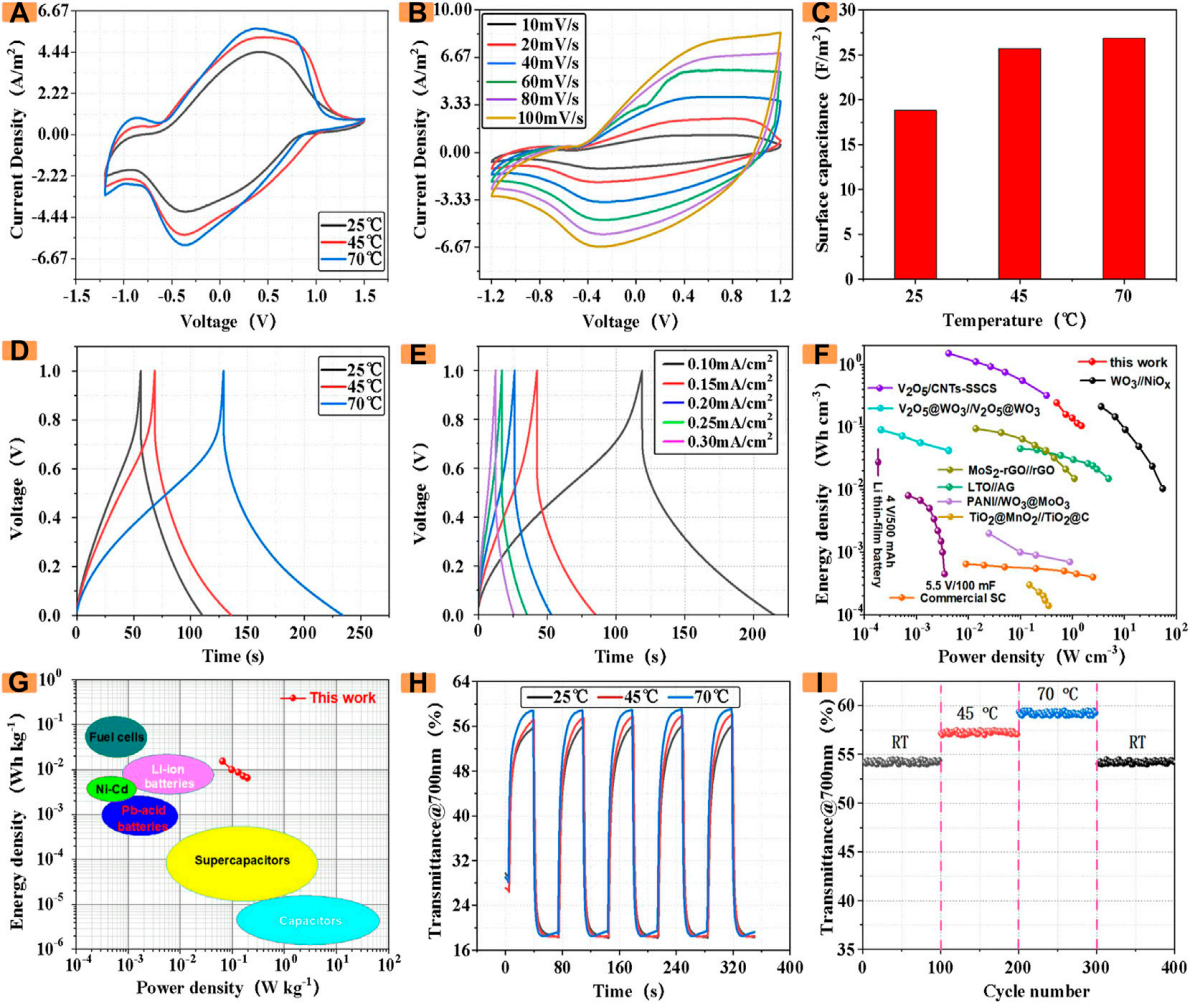
**(A)** CV at different temperatures **(B)** CV curves at different scan rates **(C)** Influence of temperature on area capacitance **(D)** GCD at different temperatures **(E)** GCD profiles at various current densities **(F,G)** Ragone plots in compared with other similar works and some representative energy storage devices. **(H)** Influence of temperature on the transmittance of devices **(I)** Cycling stability at progressively varied temperatures.

As a reliable ECD, the stability under high temperature environment is also of great significance to its application. Therefore, the electrochemical and optical properties of ECDs containing different CDs were analyzed. The following only shows the electrochemical and optical properties of ECDs with 20 mg of CDs introduced at different temperatures. According to the study of ECDs at different temperatures, we found that the ECDs with CDs still maintained relatively stable electrochemical and optical properties at different temperatures. As shown in Figure 6A, with the increase of temperature, the envelope area of the CV (50 mV/s) with the masses of CDs gradually increases.

As a reliable ECD, the stability under high temperature environment is also of great significance to its application. Therefore, the electrochemical and optical properties of ECDs containing different CDs were analyzed. The following only shows the electrochemical and optical properties of ECDs with 20 mg of CDs introduced at different temperatures. According to the study of ECDs at different temperatures, we found that the ECDs with CDs still maintained relatively stable electrochemical and optical properties at different temperatures. As shown in Figure 6A, with the increase of temperature, the envelope area of the CV (50 mV/s) of ECD with the introduction of CDs gradually increases. Furthermore, the electrochemical impedance spectroscopy (EIS) measurement was performed at room temperature (Supplementray Figure S6).

Figure 6B shows the CV curve of ECDs prepared at different scan rates (10–100 mV/s). It can be seen from the figure that even at high scan rates of 100 mV/s, it can still maintain a relatively stable shape, which indicates that ECD has good charge and discharge performance. In order to quantitatively analyze the change of the area capacitance of the device at different temperatures, we used an electrochemical workstation to measure the capacitance of the ECD introduced with CDs, as shown in Figure 6C. The areal capacitance of the color-changing device also increases significantly, which indicates that the ECD incorporating CDs still maintains stable electrochemical performance in a high-temperature environment.

Subsequently, the GCD of the ECD incorporating CDs was investigated at a current density of 0.1 mV/cm^2^. As shown in Figure 6D, at room temperature of 25°C, the charge and discharge time was about 110 s. As the temperature increased, the time of charging and discharging once reached 230 s when the temperature was 70°C. The results show that as the temperature increases, the charging and discharging time becomes longer, and the capacitance also increases gradually.

Subsequently, We investigated GCD with an ECD containing 20 mg of CDs at 25°C (Figure 6E), which were demonstrated by GCD at different current densities. ECDs with CDs can work stably in different voltage windows.

Importantly, power/energy densities are key parameters to evaluate the application potentials of energy storage devices. The power/energy densities values of the as-fabricated ECDs with CDs at different current densities (based on the total effective volume of the whole device), as well as the corresponding Ragone plots are presented in Figure 6F. As can be seen, the ECDs with CDs can deliver an energy density of 0.207 Wh cm^−3^ at 2.16 W cm−^3^. The power/energy output of the ECD with CDs is comparable to some ECDs, such as WO3@C//NC (Xu et al., 2016), MoO3//LiMn2O4 (Tang et al., 2012), NGC//LiMn2O4 (Xue et al., 2015), NBC//LiMn2O4 (Li et al., 2019), rGO@VO2//AC (Sahoo et al., 2019), Nb2O5//AC (Deng et al., 2017), Graphite//AC (Koo, Y et al., 2019), NHCS//ANHCS (Qiu et al., 2019) and so on.

Additionally, our ECDs with CDs fill the gaps between supercapacitors and Li-ion batteries, achieving high energy/power densities through integrating the merits of supercapacitors and batteries (Figure 6G).

As an optically stable ECD, the analysis of its optical properties at different temperatures is also of great significance. In this case, we investigate and analyze the *in-situ* transmittance of the ECD at different temperatures. As shown in Figure 6H, in the normal temperature environment, the transmittance of the 700 nm wavelength light is 18.3% when it is colored, and the transmittance is 55.9% in the faded state, and its optical contrast is 37.6%. As the temperature increases, the optical modulation amplitude also increases. At 70°C, the transmittance when the color fades is 58.9%, and the optical modulation amplitude also becomes the original 108%. Significantly, as shown in Figure 6I, ECDs with CDs also exhibits impressive temperature adaptability. Although experiencing 400 repetitive heating/cooling cycles, when the temperature again drops to room temperature, the ΔT of the as-obtained ECD can still maintain 97.4% of its initial value, implying that superior electrochromic performance and outstanding temperature-resistive long-term cycle stability. The experimental results show that the ECDs incorporating CDs still have good electrochromic performance in different temperature environments.

## Summary

In summary, an all-solid-state smart ECD is successfully fabricated by employing MnO_2_ and PB as complementary electrochromic positive and negative electrodes meanwhile by introducing CDs into the ions electrolyte layers. Compared with traditional electrochromic devices (ECDs), ECDs based on CDs can show a variety of different colors through the superposition of electrolyte and electrochromic material colors. Moreover, their response speed and cycle stability are better than traditional ECDs. Additionally, the resultant ECD can not only provide higher energy/power density but also present outstanding electrochemical thermal stability. The fabricated ECD can still maintain a stable and reversible color change even in harsh environments where operating temperatures are constantly changing from RT to 70°C. This research presents a flexible strategy to design and fabricate multicolor ECDs for intelligent displays and electronics with tunable transparency and colors.

## Data Availability

The original contributions presented in the study are included in the article/Supplementary Material, further inquiries can be directed to the corresponding authors.
