# Supplementary material for: Towards two-dimensional color tunability of all-solid-state electrochromic devices using carbon dots
Source: Front Chem. 2022 Aug 30;10:1001531. doi: 10.3389/fchem.2022.1001531 (PMC9468610; doi:10.3389/fchem.2022.1001531)
Supplement: Supplementary file 1 [file DataSheet1.PDF]

Supporting Information

Towards Two-dimensional Color Tunability of All-solid-state Electrochromic Devices Using Carbon Dots

Chen Li[1,2,3], Mingshuo Zhen[1,2], Boshan Sun[1,2,3], Yingping Hong[1,2,3*], Jijun Xiong[1,2,3], Wenzhi Xue[1], Xiaohua Li[4], Zhongkun Guo[2], Lei Liu[4*]

[1]Science and Technology on Electronic Test and Measurement Laboratory, North University of China, Taiyuan, China

[2]State Key Laboratory of Dynamic Measurement Technology, North University of China, Taiyuan, China

[3]Key Laboratory of Instrumentation Science & Dynamic Measurement, Ministry of Education, North University of China, Taiyuan, China

[4]School of Energy and Power Engineering, North University of China, Taiyuan, China

*** Correspondence:**

Lei Liu, Yingping Hong

liulei91@nuc.edu.cn, hongyingping@nuc.edu.cn

Content of Supporting Information

1. Heating treatment program of MnO_2 electrode
2. Ex-situ XPS patterns of the MnO_2 electrode
3. The thicknesses of the MnO_2 films
4. TEM of carbon dots（CDs）
5. Schematic about the *in-situ* measurement of devices
6. Electrochemical impedance spectroscopy behaviors of the electrochromic device (ECD) with CDs.

1. Heating treatment program of MnO_2 electrode

The MnO_2 film was prepared by electrodeposition on the surface of transparent Indium-Tin-Oxide (ITO) glass substrates at a current of 2.5 mA for 3 min in a three-electrode system. Then, as-fabricated films were heated at 180 °C (MnO_2) for 2 h in Ar atmosphere and then followed by slow cool down to room temperature. Here, annealing process was divided into three steps of the heating up, thermal retardation and cooling.

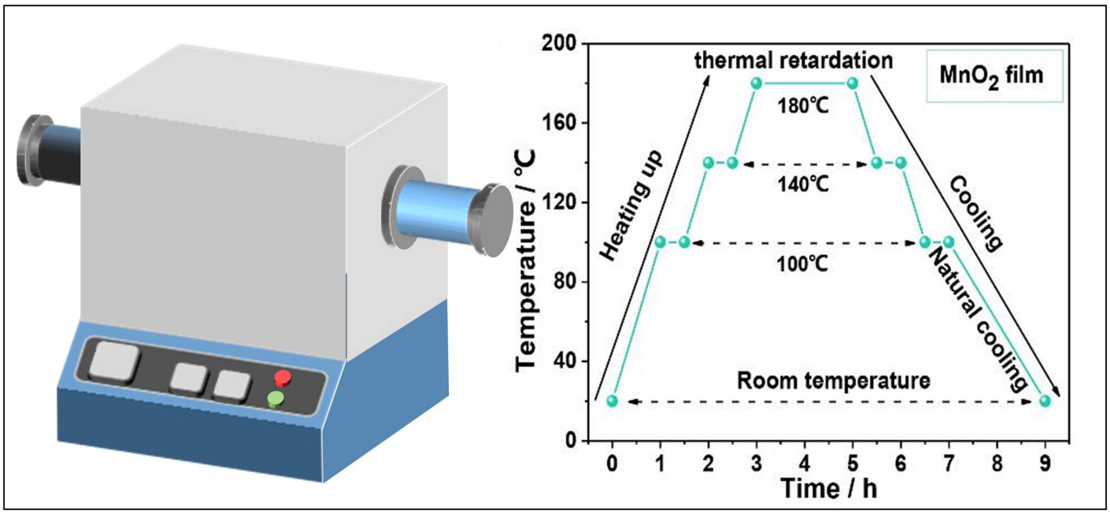

Figure S1. The annealing parameters of the MnO_2 films.

2. Ex-situ XPS patterns of the MnO_2 electrode

XPS survey spectrum was investigated to demonstrate the existence of MnO_2 and show the chemical composition, which all peaks of Mn, C, O elements are clearly observed after calibrating the peak of C1s (**Figure S2a**). After charging, the amount of Mn^{4+}, Mn^{3+} and Mn^{2+} are about 59.6%, 32.1% and 8.3%, respectively (**Figure S2b, bottom**). After discharging, the amount of Mn^{4+}, Mn^{3+} and Mn^{2+} are about 33.8%, 40.5% and 25.7%, respectively (**Figure S2b, top**). Hence, there is the reversible transition of Mn valence states during the charging/discharging process in Li^+ electrolyte.

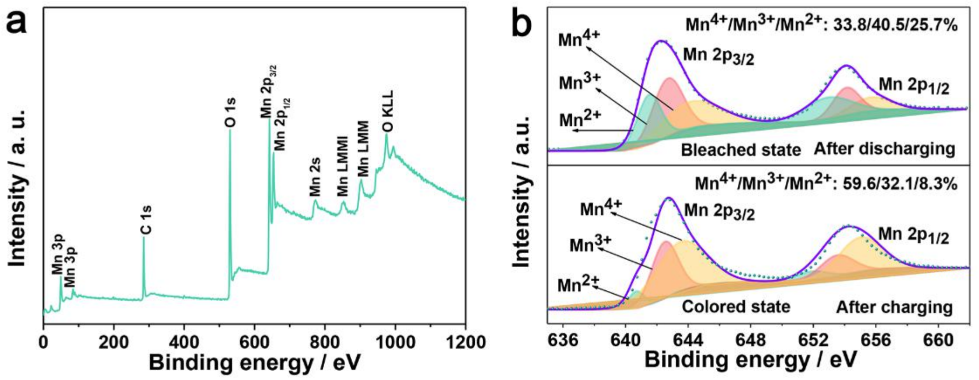

Figure S2. Ex-situ XPS patterns of the MnO_2 electrode. (a) XPS pattern. (b) HR-XPS Mn2p patterns.

3. The thicknesses of the MnO_2 films

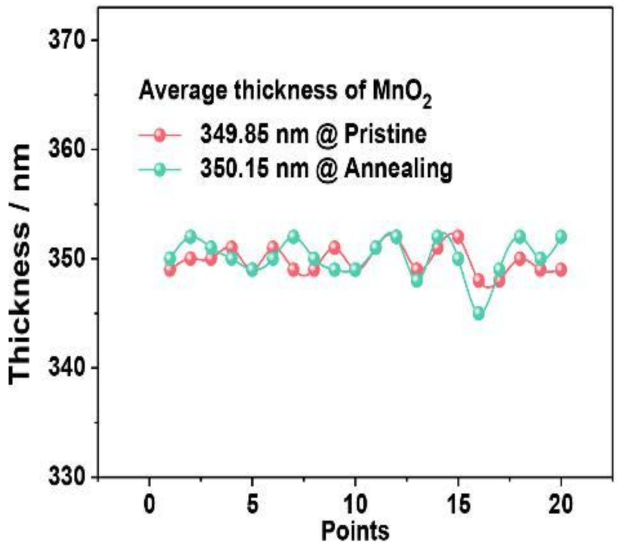

Figure S3. The thicknesses of the MnO_2 films.

4. TEM of carbon dots（CDs）

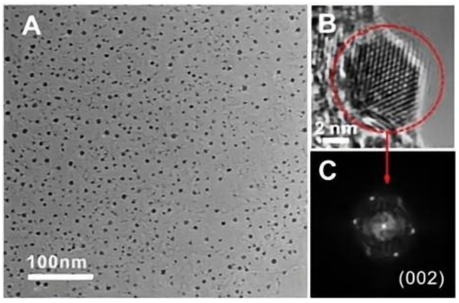

Figure S4 The structure of crystalline C-Dots. (A) TEM image of C-Dots. (B) High-resolution TEM image and (C) SAED pattern of a single C-Dot particle.

5. Schematic about the *in-situ* measurement of devices

As shown in **Figure S5**, the ECD was fixed to a ceramic heating chip that contained holes for the high-temperature measurements.

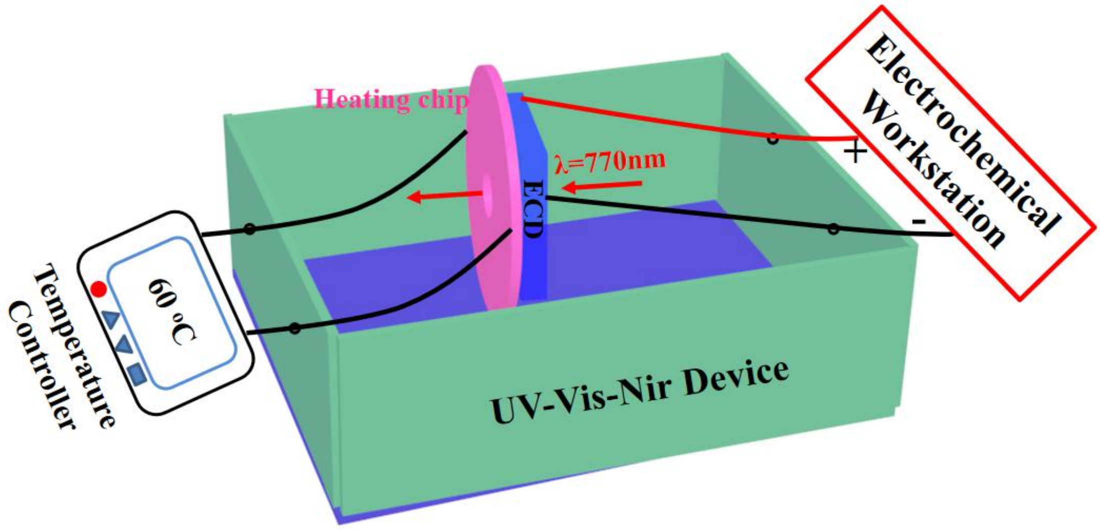

Figure S5. Schematic about the *in-situ* measurement of devices.

6. **Electrochemical impedance spectroscopy behaviors of the ECD with CDs**

The electrochemical behavior of the ECD with CDs was further estimated by using electrochemical impedance spectroscopy (EIS) at the fixed potential of 0.3V with a perturbation of 10 mV from 10^{-2} to 10^{5} Hz (**Figure S6**).

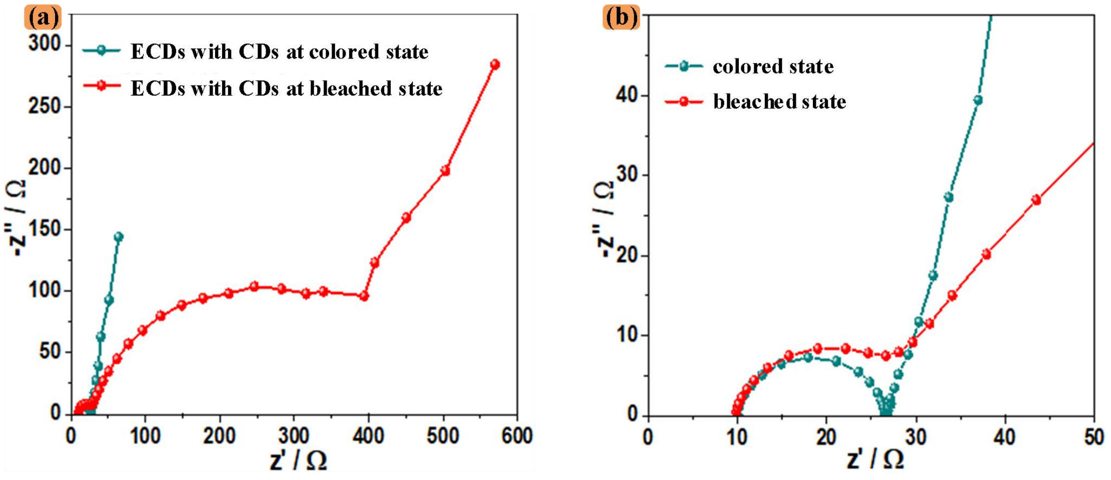

Figure S6. The EIS curves of the ECD with CDs range from 10^{-2} to 10^{5} Hz. (a) The original image (b) Partially magnified image
